# Application of natural zeolite adsorption in cooperation with photosynthesis for the post-treatment of microbial fuel cells†

**DOI:** 10.1039/d4ra04672b

**Published:** 2024-08-22

**Authors:** Que Nguyen Ho, Taira Hidaka, Mukhlis A. Rahman, Naoko Yoshida

**Affiliations:** a Department of Civil Engineering, Nagoya Institute of Technology Nagoya Japan yoshida.naoko@nitech.ac.jp; b Department of Environmental Engineering, Graduate School of Engineering, Kyoto University, Kyoto University Katsura Nishikyo Kyoto 615-8540 Japan; c Advanced Membrane Technology Research Centre (AMTEC), School of Chemical and Energy Engineering (FCEE), Universiti Teknologi Malaysia 81310 UTM Skudai Johor Malaysia

## Abstract

Microbial fuel cells (MFCs) are a promising technology that directly converts organic matter (OM) in wastewater into electricity while simultaneously degrading contaminants. However, MFCs are insufficient for the removal of nitrogenous compounds. Therefore, the post-treatment of MFCs is essential. This study was the first to use natural zeolite adsorption integrated with photosynthesis (ZP) for post-treating MFCs. In this system, no external energy was required; instead, natural light was used to promote the growth of photosynthetic microorganisms, thereby enhancing contaminants removal through the photosynthesis process. To assess the effectiveness of the method, comparisons were conducted under two conditions: dark (no photosynthesis) and light (with photosynthesis). In darkness, extending hydraulic retention time (HRT) enhanced COD and BOD removal by 19.8% and 28.9%, respectively. When exposed to natural light, improvements were even more notable, with COD and BOD removal reaching 32% and 40%, respectively. In both conditions, the method effectively removed NH_4_^+^, achieving 60% efficiency in darkness and 84.5% in light. This study showed that the adsorption capacity of the zeolite reached saturation when the cumulative liquid volume per unit weight of the zeolite exceeded 0.2 L g^−1^. The key functional photosynthetic microbes were investigated using 16S rRNA and 18S rRNA. This revealed the presence of microorganisms such as *Chlorobium*, *Acidovorax*, *Novosphingobium*, and *Scenedesmus*, which likely play a role in enhancing the efficiency of photosynthesis in removing contaminants. The study findings indicated that the integration of MFCs-ZP represents an eco-friendly approach capable of resource recovery from wastewater while also meeting discharge standards.

## Abstract 2

Natural zeolites based on adsorption, combined with photosynthesis as secondary treatment for microbial fuel cells, achieved 84.5% removal of NH_4_^+^.

## Graphical abstract

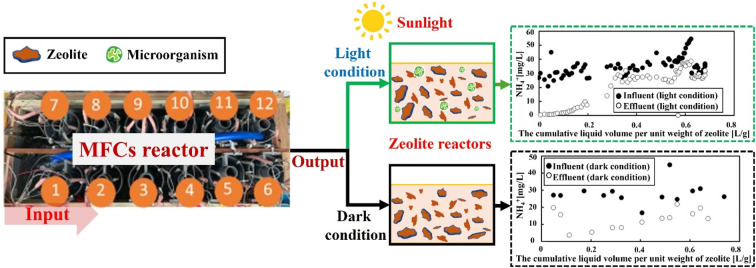

## 1. Introduction

Wastewater treatment (WWT) is essential to preserve the environment by mitigating water pollution. However, WWT is a process that requires a significant amount of energy. For instance, earlier research indicated that the electricity consumption for WWT accounted for 4% in the USA, 0.7% in China, and over 1% in Europe.^1^ Consequently, enhancing the energy efficiency of wastewater treatment plants is pertinent when considering both economic and environmental aspects.^2^ Hence, current innovations in WWT technologies are not only aimed at improving contaminant removal efficiency but also at reducing energy consumption. Among these technologies, microbial fuel cells (MFCs) have emerged as environmentally friendly electrical devices and have quickly evolved into sustainable systems. This is attributed to their capacity to simultaneously treat wastewater and generate energy.^3^ In MFCs, the oxidation of organic matter (OM) in wastewater produces electrons and protons in the anode compartment *via* exoelectrogens. Protons and electrons are transferred to the cathode chamber by the membrane and electrodes, respectively. These processes lead to the production of both clean water and energy.^4^ However, in certain cases, the application of MFCs technology alone does not meet the nitrogen effluent quality requirements^5,6^ because of the lack of oxidants, such as oxygen for nitrification and NO_2_^−^ for the anammox process, in the anolyte in MFCs. In certain MFCs, ammonia removal is observed, but primarily in specific types that apply a gas diffusion membrane (GDM) or cation exchange membrane (CEM) to separate the anolyte and air. In both cases, the major mechanism involves the vaporization of the ammonia generated through the reduction of ammonium on the cathode.^7–9^ MFCs with GDM facilitate oxygen diffusion to the anolytes and enhance nitrification.^10,11^ However, MFCs equipped with anion exchange membranes (AEM) were superior in electricity generation owing to the migration of pH imbalances, as opposed to CEM and GDM. These facts highlight the trade-off between ammonia removal and electricity.

The problem with ammonia removal is that the nitrification and anammox processes require oxidants. The dissolved oxygen (DO) concentration must exceed 0.5 mg L^−1^, the minimum concentration required for nitrite oxidation during nitrification.^12^ Intermittent aeration in MFCs partially reduces ammonia, enabling an increase in ammonia oxidizers and anammox despite the low removal efficiency.^13^ The exceptionally high anode potential can enable the anammox process in MFCs, although powerful oxidants, such as potassium persulfate, must be provided in the cathode.^14^ Alternative approaches include adsorption and indirect oxygen supplementation through photosynthesis. Two representative processes, adsorption^15,16^ and photosynthesis^17^ have been developed because of their ease of operation, cost-effectiveness, and high nitrogen removal efficiencies.

Adsorption is a mass transfer process in which substances accumulate at the interface of two phases, liquid–liquid or liquid–solid, and the adsorbing material is called an adsorbent.^18^ Hence, the effectiveness of adsorption in wastewater treatment relies on the characteristics of the adsorbent, such as negative electric charge, high surface area, and micro-/mesoporous properties, for the efficient removal of contaminants.^19^ Natural zeolites stand out because of their various advantages, particularly their roles as ion exchangers, catalysts, and adsorbents.^20^ Natural zeolites have a porous structure that can accommodate a wide variety of cations such as Na^+^, K^+^, Ca^2+^, Mg^2+^, and others. These positive ions are loosely bound and can be easily exchanged for others in the contact solution.^21^ This makes it an attractive approach for pollutant removal.^22–24^ Furthermore, zeolites are natural hydrated aluminosilicate materials with a high affinity for NH_4_^+^,^25^ potential for removal NH_4_^+^ from wastewater. Additionally, natural zeolites have a lower cost per gram of nitrogen removed compared to other polymeric cation exchange resins.^26^ For example, previous studies have indicated that zeolite eliminates approximately 90% NH_4_^+^ in industrial wastewater,^27^ as well as 82.97% of ammonia in synthetic wastewater.^23^ Additionally, Han *et al.* demonstrated that a constructed wetland incorporating zeolite as a substrate could significantly remove NH_4_^+^ and total nitrogen (TN) with percentage of 72.99%, and 70.71%, respectively, from swine wastewater.^28^

Oxygenic photosynthetic microorganisms generate oxygen *via* photosynthesis and have great potential for treating various nitrogen-contaminated wastewaters.^17,29^ These microorganisms can metabolize substrates such as nitrate and ammonia in the presence of light or oxygen.^29^ To date, numerous studies have employed photosynthetic microorganisms for removal nitrogen from various wastewaters: 83.2% of ammonia removal efficiency in chicken manure wastewater,^30^ 90–94% of total nitrogen removal efficiency in poultry processing wastewater,^31^ 72–98% of ammonia removal efficiency in synthetic wastewater.^29^

Therefore, zeolite adsorption and photosynthetic microorganisms are advantageous for nitrogen removal during wastewater treatment. However, earlier research primarily focused on eliminating nitrogen and OM from raw wastewater, as specifically mentioned in studies.^32–34^ This type of wastewater contains high concentrations of contaminants, which can positively affect the removal efficiency. Additionally, Meng Wang and co-authors employed natural zeolite and the photosynthetic microorganism *Chlorella* (comprising over 95% of the total cells) together to treat high-strength ammonium wastewater (1180 mg L^−1^ NH_4_^+^). In this study, the authors utilized artificial LED lights to promote algae growth.^26^ In our work, we utilized natural zeolites in cooperation with photosynthesis for nitrogen removal from wastewater as a secondary process, incorporated into MFCs. This involved lower concentrations of contaminants (*i.e.*, OM and NH_4_^+^). We used natural light to promote the growth of photosynthetic microorganisms in the wastewater itself. To the best of our knowledge, there is limited research on this approach. The findings of this study show the significant potential for offering environmentally friendly solutions to effectively eliminate contaminants from wastewater, particularly nitrogen, when combined with MFCs. In this study, sewage was treated with MFCs, and the MFCs effluent was pumped into a reactor containing zeolite under both dark and light conditions to assess the effectiveness of photosynthetic microorganisms.

## 2. Material and methods

### 2.1. MFCs set-up experiment

**Fig. 1:**
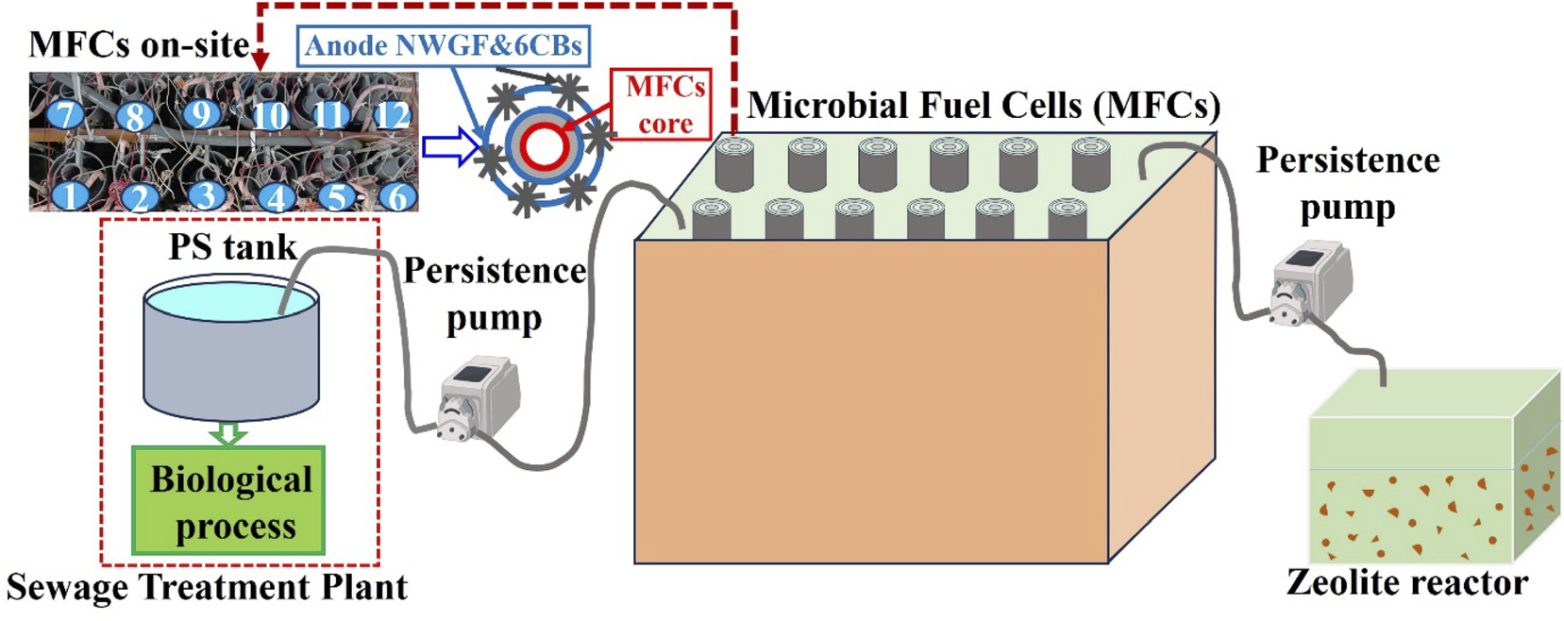
Schematic diagram illustrating the experiment setup for natural zeolite adsorption and photosynthesis. Numbers from 1 to 12 represent the 12 MFC units. PS: primary settling tank, NWGF: nonwoven graphite fabric, and CB: carbon brushes.

Fig. 1 shows a schematic diagram of zeolite adsorption for treatment of MFCs effluent. In this study, 12 units of MFCs reactors were used, each with a tubular structure with a diameter of 5 cm and a length of 100 cm. It features an air core and is equipped with a carbon-based cathode, an anion exchange membrane, a nonwoven graphite fabric anode, and five cylindrical anodes (4 cm in diameter and 100 cm in length). These anodes were constructed by bundling carbon fibers (T300B-3k-40B, Toray, Tokyo, Japan) with a stainless-steel wire and positioned around the core of the MFCs. The MFCs system was installed at the Ueda Water Treatment Center in Nagoya, Japan, which collects sewage from Nagoya City. Table 1 shows the quality of MFCs effluence, post treatment of the effluent involved zeolite adsorption and microbial photosynthetic as zeolite reactor was illuminated under natural daylight, which will be discussed in more detail in the following section. Natural zeolite, purchased from Shin Tohoku Chemical Industries (Miyagi, Japan), was used as the model adsorbent particle in the experiments. The particle size of natural zeolites has been reported to be 4–8 mm.

**Table 1:** Average composition of the effluent sewage from MFCs, utilized to influence zeolite reactors

Parameters (mg L^−1^)	Conditions		
	Dark 1	Dark 2	Light
Chemical oxygen demand (COD)	53.4	69.9	62.0
Biological oxygen demand (BOD)	14.8	20.4	18.0
NH_4_^+^		29.6	34.7
PO_4_^+^		6.1	6.3

### 2.2. Zeolite adsorption and microbial photosynthesis

#### 2.2.1. Dark zeolite champers

A cylindrical zeolite tank was devised to evaluate two distinct dark conditions characterized by variations in size and flow rate. For dark condition 1, the tank was measuring 20.3 cm in internal diameter and 51.3 cm in height and was constructed using a VU200 PVC pipe. The reactor was filled with 10 kg of natural zeolite. The effective tank capacity was 19.4 L, of which 16.6 L were taken up by zeolite and liquid, with the liquid itself occupying a volume of 12.3 L. MFCs-treated water was supplied into the tank through a bottom inlet, with an upward flow pattern, while the zeolite adsorption tank received the flow from the top. The flow rate of the liquid feed was maintained at 85 mL min^−1^ (linear velocity ∼ 0.35 cm min^−1^, hydraulic retention time (HRT) = 2.4 h). Under dark condition 2, the tank had an internal diameter of 5.1 cm, a height of 80 cm, and an empty volume of 1.63 L. We introduced 1 kg of natural zeolite, which occupied 0.43 L within the tank. The flow rate was reduced to 6.5 mL min^−1^ (linear velocity of ∼0.32 cm min^−1^, HRT = 3.6 h).

#### 2.2.2. Transparent zeolite chambers with light condition

A transparent zeolite tank was created by loading an acrylic water tank with transparent walls, measuring 40 cm in width, 10 cm in depth, and 60 cm in height, with natural zeolite filled to a height of 31 cm, totaling 8 kg in weight. MFCs-effluence was supplied from the bottom inlet, while the treated water was discharged from an upper outlet positioned at a height of 55 cm from the base of the tank. The device had a total volume of 19.5 L, with a combined liquid volume of 17.9 L, and a portion filled with zeolite having a liquid capacity of 8.9 L. The flow rate of the incoming fluid ranged from 10 to 20 mL min^−1^, resulting in HRT of 7.5 to 15 h.

### 2.3. Measurement of NH_4_^+^ and PO_4_^3−^

NH_4_^+^ concentration was assessed using an ion meter (TiN-9001, Toko Kagaku Kenkyusho, Tokyo, Japan). Standard solutions of NH_4_^+^–N at concentrations of 10 mg L^−1^ and 100 mg L^−1^ were prepared using ammonium chloride. To these solutions, 10 mL L^−1^ of NaOH solution (v : v= 1 : 1) was added to adjust the pH to 12 for both the standard solutions and samples. The NH_4_^+^ concentration was determined using a calibration curve. The concentration of PO_4_^3−^ was measured using a digital tester (HI 717, Hanna Instruments, Chiba City, Japan).

### 2.4. Measurement of COD and BOD

In this study, the COD and BOD were measured to evaluate the degradation of organic matter (OM). These samples were periodically collected from the influent and effluents of the reactor under both dark and light conditions. The analyses were performed by Toa Environmental Services Co. in Nagoya, Japan. For the analysis of COD and BOD, 15 mL and 200 mL samples were collected, respectively. Both types of samples were stored at −20 °C prior to analysis.

### 2.5. Microbial analysis

Biomass samples were collected from various locations within the reactor to study the microbial community. These locations included areas adhered to the walls of the zeolite reactor (Zeo-S), those attached to plates submerged in the reactor (Zeo-P), those affixed to the zeolite material (Zeo-Z), and those influencing the zeolite reactor (referred to as Zeo-in). These biomass samples were carefully preserved in 500 mL sterilized polyethylene containers and immediately transported to the laboratory. Upon arrival, they were stored at a temperature of 4 °C.

Microorganisms were identified using 16S and 18S rRNA sequencing analysis with a high-throughput Illumina sequencing technique (Illumina MiSeq, Illumina Inc.). Polymerase chain reaction (PCR) amplification was performed using specific primer sets (Table 1) to amplify the 16S rRNA gene (V3–V4 region) and the 18S rRNA gene (V7–V8 region). Each amplification reaction was performed in triplicate, and the primer sequences used in this study are listed in Table 2. Furthermore, the microbial community was analyzed using Qiime2. In addition to Qiime2, the SILVA reference database was used to align sequences, taxonomic classification, and phylogenetic analysis of both 16S rRNA and 18S rRNA sequences. The gene sequence data were deposited in the DNA Data Bank of Japan under accession number DRA018819.

**Table 2:** Gene primers applied for high-throughput sequencing analyses

Target genes	Primer	Sequence 5′
16S rRNA (V3–V4)	341F	TCGTCGGCAGCGTCAGATGTGTATAAGAGACAGCCTACGGGNGGCWGCAG
	805R	GTCTCGTGGGCTCGGAGATGTGTATAAGAGACAGGACTACHVGGGTATCTAATCC
18S rRNA (V7–V8)	1183F	TCGTCGGCAGCGTCAGATGTGTATAAGAGACAGAATTTGACTCAACACGGG
	1631R	GTCTCGTGGGCTCGGAGATGTGTATAAGAGACAGTACAAAGGGCAGGGACG

## 3. Results and discussion

### 3.1. Organic matter removal

**Fig. 2:**
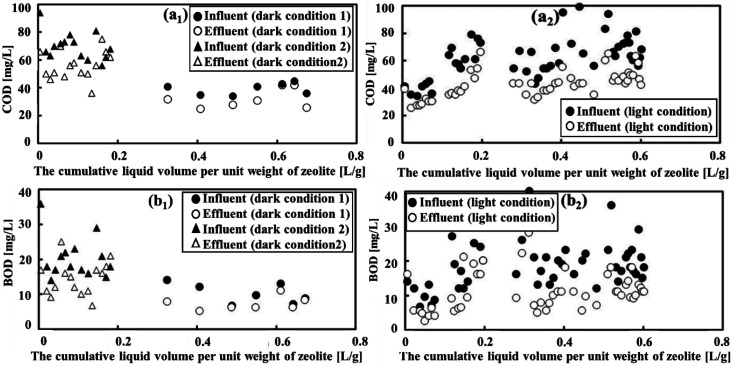
The effectiveness of COD and BOD removal under both dark and light conditions. The parameters (a_1_) and (a_2_) represented COD removal efficiency, while (b_1_) and (b_2_) indicated BOD removal efficiency. Due to variations in reactor volumes and the quantity of zeolite used, we opted to measure the cumulative wastewater amount per unit of zeolite, rather than monitoring OM removal over time. This approach facilitated the comparison of efficiency across different reactors. These values were consistently utilized in all subsequent figures.

The assessment of treatment efficiency in a wastewater treatment plant (WWTP) is based on the effective removal of biodegradable and nonbiodegradable organic compounds, with COD and BOD serving as important parameters. In dark condition 1, low degradation of COD and BOD was observed, with average COD degradation from 53.4 mg L^−1^ to 15.9 mg L^−1^ and BOD degradation from 14.8 mg L^−1^ to 13.6 mg L^−1^ (Fig. 2(a1) and (b1)), corresponding to removal efficiencies of 15.9% and 8.14%, respectively. However, degradation increased when transitioning to condition 2, with the average COD and BOD decreasing from 69.9 mg L^−1^ to 56.1 mg L^−1^ and 20.3 mg L^−1^ to 14.5 mg L^−1^, respectively (as seen in Fig. 2(a_1_) and (b_1_)), resulting in a higher percentage removal of both parameters. Notably, the BOD removal efficiency increased to 28.9%, whereas the COD removal efficiency showed a slight increase to 19.8%.

In darkness, the OM is considerably reduced owing to adsorption on the surface of the zeolites, as discussed in previous studies.^35,36^ The enhanced removal efficiencies of COD and BOD under dark condition 2 can be attributed to several factors. First, a decrease in the flow rate, leading to an extended HRT, increases the adsorption capacity of natural zeolite for OM. This finding aligns with prior research.^37^ Second, the high concentrations of BOD and COD had a substantial impact on the adsorption capacity of zeolite. This resulted in a noticeable improvement in the BOD and COD removal efficiencies from wastewater, as illustrated in Fig. 2(a_1_) and (b_1_).

Conversely, when subjected to light conditions, there was a substantial increase average degradation of both COD (from 62.4 mg L^−1^ to 42.6 mg L^−1^) and BOD (from 18.3 mg L^−1^ to 11.0 mg L^−1^) (refer to Fig. 2(a_2_) and (b_2_)), indicating removal efficiencies reaching 31.7% and 40.0%, respectively. These findings can be attributed to the increase in the number of photosynthetic microorganisms in the reactor under natural light illumination. The presence of light promoted the growth of both bacteria and microalgae,^38^ which engage in metabolic processes that enhance the degradation of OM. In the reactor, microalgae absorb CO_2_ and organic pollutants through photosynthesis, releasing O_2_ and producing OM. Simultaneously, bacteria consume oxygen and OM through respiration, creating a cycle of CO_2_ and O_2_ between the algae and bacteria. This collaboration between algae and bacteria enhances the efficiency of wastewater treatment, particularly in removing OM.^39^ The specific characteristics of these microorganisms are discussed in the following sections.

**Fig. 3:**
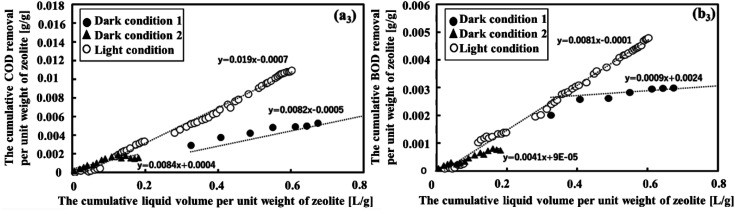
The relationship between cumulative COD and BOD removal per unit weight of zeolite and the cumulative liquid volume per unit weight of zeolite is depicted in figures (a_3_) and (b_3_), respectively.

Fig. 3 clearly illustrates the variation in COD and BOD removal efficiencies across diverse conditions. The slopes for COD and BOD were 0.019 and 0.081, respectively, and it was evident that light conditions were optimal for effectively removing both COD and BOD following MFCs treatment. This was due to the presence of both zeolite-adsorbing and OM degradation bacteria under these conditions. Furthermore, it is noteworthy that under dark condition 2, the patterns of removal of COD and BOD exhibited remarkable similarity. They increased rapidly as the cumulative liquid volume per unit weight of zeolite increased from 0.005 to 0.16 L g^−1^ before stabilizing. This suggests that the zeolite adsorption could reach its saturation point, and further increasing the wastewater feed did not significantly affect its adsorption capacity. This observation was consistent with the findings of a study by Huang *et al.*^40^ Under dark condition 1, where the slopes were modest at 0.0082 and 0.0009 for COD and BOD, respectively, the removal efficiency was relatively low. This outcome was in line with the notion that a high flow rate, leading to reduced HRT, does not allow sufficient time for the zeolite to adsorb OM. Thus, dark condition 2 performed better than dark condition 1 in terms of COD and BOD removal. As a result, dark condition 2 was chosen to assess the removal of NH_4_^+^ and PO_4_^3−^ and to make a comparison with light conditions.

### 3.2. Removal of NH_4_^+^ and PO_4_^3−^ ions

**Fig. 4:**
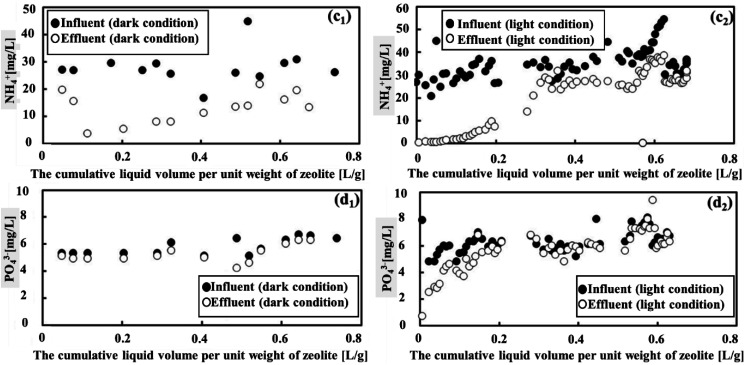
The removal efficiency of NH_4_^+^ under both dark condition 2 and light condition exhibited a trend where zeolite adsorption and photosynthetic microbes initially performed well but subsequently declined (c_1_) and (c_2_). However, it is important to note that they were not effective in removing PO_4_^3−^, regardless of the light condition (d_1_) and (d_2_).

Under both light and dark conditions, zeolite adsorption and photosynthesis proved effective in removing NH_4_^+^ but were less efficient in removing PO_4_^3−^, as shown in Fig. 4. When the cumulative liquid volume per unit weight of zeolite remained below 0.2 L g^−1^, it was the ideal condition for NH_4_^+^ removal. Within this range, NH_4_^+^ degradation displayed a high rate, with effluent concentrations of approximately 3.1 mg L^−1^ and 10.4 mg L^−1^ (as seen in Fig. 4(c1) and (c2)), corresponding to average removal efficiencies of 84.5% and 60% under light and dark conditions, respectively. The results demonstrated that the treatment system effectively removed NH_4_^+^ from sewage, showing performance comparable to the reported systems (see Table S1 in the ESI†). Thus, considering the NH_4_^+^ parameter, natural zeolite in cooperation with photosynthesis was more efficient compared to MFCs, which did not effectively remove this parameter from sewage, as shown in Fig. S1 (ESI†). Beyond this point, however, the removal efficiency began to decline. Considering the removal efficiencies of COD and BOD along with this observation, it can be concluded that the zeolite adsorption capacities reached saturation when the cumulative liquid volume per unit weight of zeolite exceeded 0.2 L g^−1^. In contrast, the degradation of PO_4_^3−^ was low in both conditions, showing average degradation declined from 5.8 mg L^−1^ to 5.4 mg L^−1^ and 6.3 mg L^−1^ to 5.7 mg L^−1^ for dark and light conditions, respectively. This was equivalent to the removal efficiency of PO_4_^3−^ of 5.54% in dark conditions and 14.9% in light conditions.

In summary, the presence of photosynthetic microorganisms under light conditions noticeably enhanced the contaminant removal efficiency such as COD, BOD, and NH_4_^+^. The variation in the contaminant removal performance between dark and light conditions highlighted the significance of photosynthesis as an environmentally friendly approach for enhancing the removal of pollutants from MFCs-treated wastewater.

**Fig. 5:**
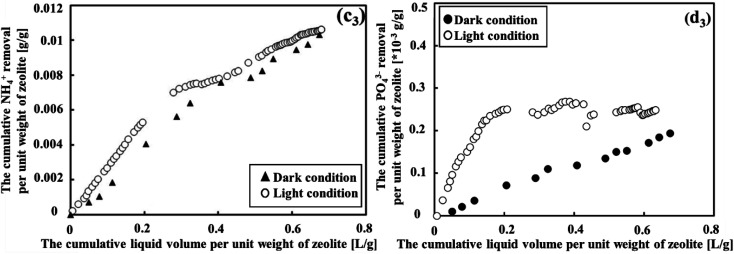
The cumulative NH_4_^+^ and PO_4_^3−^ removal per unit weight of zeolite, as a function of the cumulative liquid volume per unit weight of zeolite under dark condition 2 and light condition, is presented in (c_3_) and (d_3_), respectively.

Fig. 5 presents the contrast in the removal efficiency between NH_4_^+^ and PO_4_^3−^. Notably, cumulative NH_4_^+^ removal exhibited significant growth under both light and dark conditions. Initially, the light condition performed better than the dark condition; however, this difference diminished over time. Generally, the percentage of NH_4_^+^ removed from wastewater was relatively consistent across different conditions, with values of 43.8% under light conditions and 37.6% under dark conditions. To determine if there was a difference in efficiency between the two conditions, the Mann–Whitney *U* test was applied. The statistical test revealed a *p*-value of 0.2233, indicating that the NH_4_^+^ removal efficiency was not significantly different between dark and light conditions. In contrast, the cumulative PO_4_^3−^ removal per unit weight of the zeolite showed a slight increase. However, under light conditions, there was a more pronounced difference in cumulative PO_4_^3−^ removal compared to dark conditions. The Mann–Whitney *U* test revealed a *p*-value of 0.000174, indicating that the PO_4_^3−^ removal efficiency was significantly different between dark and light conditions. This difference could be attributed to the influence of photosynthetic microorganism activity under light condition. Therefore, in order to improve the removal of both NH_4_^+^ and PO_4_^3−^, it was considered essential to optimize the photosynthesis process.

### 3.3. Microbial community diversity analysis

**Fig. 6:**
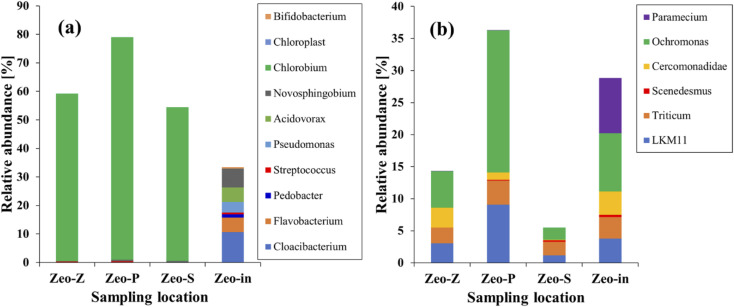
Relative abundance of the most abundant microbial community structure at the genus level detected, from 16S rRNA (a) and 18s rRNA (b) sequencing of the samples taken in the zeolite reactor under light condition.

The previous section highlighted that the efficiency of contaminant removal increased under light conditions, which could be attributed to the increased activity of microorganisms. Analysis of 16S rRNA and 18S rRNA revealed the presence of more than 10 and 6 genera, respectively, in all samples (Fig. 6). Under 16S rRNA analysis, all genera were present in the Zeo-in samples. Notably, *Chlorobium* (53.92–78.02%), *Novosphingobium* (0.11–0.45%), *Streptococcus* (0.07–0.33%), and *Cloacibacterium* (0.08–0.13%) were identified as core genera, collectively accounting for nearly 78.9% of the total sequences. Whereas almost all the sequences detected in the Geo-in samples were no longer present because *Chlorobium* had become the predominant genus. This shift can be attributed to the fact that *Chlorobium* is a photosynthetic bacterium,^41^ that outcompetes non-photosynthetic microorganisms (NPM) for nutrients and OM in wastewater. This competitive advantage resulted in a decrease in the NPM population. Furthermore, previous studies have demonstrated *Chlorobium*'s ability to remove COD from wastewater.^17,42^*Chlorobium* has also been shown to oxidize reduced-sulfur compounds.^43^ When sulfur compounds are introduced into sewage, they contribute to the overall organic load by increasing both BOD and COD in wastewater. Consequently, the reduction in sulfur compounds led to a decrease in both BOD and COD in the sewage, a trend that aligns well with the findings presented in Fig. 2(a_2_) and (b_2_).

Although dominant microorganisms such as *Chlorobium* and *Streptococcus* did not exhibit the capability to directly remove PO_4_^3−^ and NH_4_^+^, the concentrations of these substances still decreased. It was hypothesized that the microorganisms responsible for PO_4_^3−^ and NH_4_^+^ removal might still be present in the reactor, even if their sequences could not be detected. Notable examples of such microorganisms include *Acidovorax*, a genus within the family *Comamonadaceae*, which can accumulate phosphate in wastewater.^44,45^*Novosphingobium* is renowned for its ability to break down a diverse range of aromatic hydrocarbons and plays a crucial role in the biogeochemical cycles of carbon, nitrogen, and chlorine in its surrounding environments. It exhibits the capability to convert nitrate into nitrite.^46^

Furthermore, the 18S rRNA analysis revealed the presence of *Scenedesmus*, a microorganism known for its capacity to eliminate nitrogen and phosphorus from wastewater^47^ (as shown in Fig. 6b). In wastewater, *Scenedesmus* can remove NH_4_^+^ in a two-way direct utilization by itself and remove nitrogen in the form of ammonia with air employed for the aeration of the medium. In addition, it can take up phosphorus from wastewater, leading to a reduction in the concentration of PO_4_^3−^ in the effluent.^48^ Normally, microalgae such as *Scenedesmus* can store phosphorus within biomass as polyphosphates. This storage actively participates in cell metabolism or is reserved when the external PO_4_^3−^ concentration becomes limited.^49^ Hence, the removal efficiency of COD, BOD, PO_4_^3−^, and NH_4_^+^ increased under light conditions, which was clearly observed through the microorganism's activities.

### 3.4. Implication

**Fig. 7:**
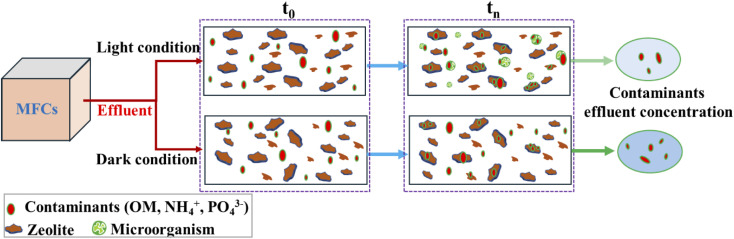
A schematic diagram illustrates the kinetics of contaminant removal by zeolite in collaboration with photosynthesis. It presents a conceptual model, depicting the general progression of the experiment from the initial stage (*t*_0_) to completion (*t*_n_).

A conceptual model was employed to understand the kinetics of contaminant removal during the post-treatment of effluent from MFCs under both dark and light conditions using zeolite in collaboration with photosynthesis, as illustrated in Fig. 7. Regardless of the conditions, natural zeolites can remove contaminants through adsorption or ion exchange. Specifically, OM can be adsorbed onto zeolite surfaces *via* an electrostatic adsorption mechanism.^34^ However, this approach appeared to be ineffective, resulting in a low OM removal efficiency under dark conditions, as depicted in Fig. 2(a_1_) and (b_1_). This is because natural zeolites demonstrate limited adsorption of organics in aqueous solutions owing to their hydrophilic surfaces.^34^ Modifying the surface with surfactants can alter its functionality by introducing hydrophobic groups, thereby enhancing the adsorption capacities for various organics.^34^ When the zeolite was exposed to natural light, as proposed in this work, it exhibited enhanced efficiency in removing OM through photosynthesis, as depicted in Fig. 2(a_2_), (b_2_) and 3. In this study, due to the lack of nitrifying microbes detected in the microbial analysis of the reactor, the primary mechanism for NH_4_^+^ removal from the wastewater was hypothesized to be ion exchange. In this process, NH_4_^+^ is taken up by the zeolite through exchange with Na^+^, Ca^2+^, and K^+^ ions. Normally, Ca serves as the secondary primary cation in the crystal framework of natural zeolites, constituting 2.09% of the total atomic weight. Ca can be readily replaced by NH_4_^+^ in the solution.^33^ Previous research indicates that the adsorption capacity of natural zeolites for NH_4_^+^ ranges from 2.7 to 30.6 mg g^−1^.^34^ It has been reported that Ca^2+^ is released during the exchange process with NH_4_^+^, and in the presence of PO_4_^3−^ in the solution, it precipitates as Ca_3_(PO_4_)_2_ on the zeolite surface. This process contributes to the removal of phosphorous from wastewater, as described by reactions (1) and (2).^32,33^ However, this study revealed a low efficiency in removing PO_4_^3−^, which is potentially influenced by competitive ion exchange between Ca^2+^ and NH_4_^+^ with another zeolite cation, leading to a reduction in the concentration of Ca^2+^ in the solution. Additionally, other factors, such as pH, can affect both the release of Ca^2+^ and the formation of Ca_3_(PO_4_)_2_ in solution.^32,33^ Hence, additional research is needed to enhance the removal of phosphate from wastewater using natural zeolites in collaboration with photosynthesis, with a significant concern regarding pH conditions.

1 Zeolite–Ca^2+^ + 2NH_4_^+^ → zeolite–2NH_4_^+^ + Ca^2+^

2 Ca^2+^ + PO_4_^3−^ → Ca_3_(PO_4_)_2_↓

Therefore, the primary processes for removing contaminants from wastewater under dark conditions are adsorption and ion exchange. Additionally, under these conditions, a prolonged HRTs enhanced the interaction time between OM and zeolite, facilitating the removal of OM through adsorption. This was demonstrated under dark condition 2 (Fig. 2(a_1_) and (b_1_)). In contrast, under light conditions, the presence of microorganisms such as *Chlorobium*, *Acidovorax*, *Novosphingobium*, and *Scenedesmus* contributed to the removal of contaminants, as described in the previous section. Hence, utilizing zeolites in conjunction with photosynthesis represents a novel approach that offers advantages, such as cost-effectiveness and environmental friendliness. Furthermore, this approach addresses the limitations of MFCs in terms of nitrogen removal. In summary, this technique holds promise not only for MFCs but also for the secondary stages of other methods aimed at enhancing nitrogen and OM removal in effluents.

## 4. Conclusion

The findings revealed that factors such as flow rate and HRT influence contaminant concentrations, and the activity of photosynthetic microorganisms impacted the removal efficiency of COD, BOD, PO_4_^3−^, and NH_4_^+^. The key findings are as follows.

(1) Under dark conditions, a decrease in the flow rate combined with an increase in HRT resulted in improved removal efficiency for COD and BOD. This suggests that the contact time between OM and zeolite played a significant role in zeolite's adsorption of OM, leading to increased removal of OM from sewage.

(2) Under light conditions, the activities of microorganisms, such as *Chlorobium, Acidovorax, Novosphingobium*, and *Scenedesmus* appeared to contribute to a reduction in the concentrations of COD, BOD, PO_4_^3−^ and NH_4_^+^. Although the removal efficiency for PO_4_^3−^ was relatively low, the heightened activity of microorganisms under light condition created a substantial difference in PO_4_^3−^ removal between light and dark conditions.

These findings improve our understanding of the effectiveness of zeolite in conjunction with photosynthesis in eliminating OM and nutrients from MFCs effluents. Combining these methods is an ideal approach for reducing the carbon footprint of wastewater treatment systems. However, this system was unable to remove PO_4_^3−^ from sewage. Therefore, further research is needed to enhance the system's efficiency to effectively address all contaminants.

## Data availability

Data for this article are available at https://figshare.com/account/home at https://doi.org/10.6084/m9.figshare.26113792.

## Author contributions

Q. N. H.: methodology, formal analysis, data curation, writing-original draft, software, visualization, writing-review & editing. T. H.: writing-review & editing. M. A. R.: writing-review & editing. N. Y.: conceptualization, methodology, writing-review & editing, supervision, funding acquisition, project administration.

## Conflicts of interest

There are no conflicts to declare.

## Supplementary Material

RA-014-D4RA04672B-s001
