# Supplementary material for: Application of natural zeolite adsorption in cooperation with photosynthesis for the post-treatment of microbial fuel cells
Source: RSC Adv. 2024 Aug 22;14(36):26484–93. doi: 10.1039/d4ra04672b (PMC11339683; doi:10.1039/d4ra04672b)
Supplement: RA-014-D4RA04672B-s001 [file RA-014-D4RA04672B-s001.pdf]

Supplementary Information (SI) for RSC Advances.
This journal is © The Royal Society of Chemistry 2024

SUPPLEMENTARY INFORMATION

Application of natural zeolite adsorption in cooperation with photosynthesis for the post-treatment of microbial fuel cells

Que Nguyen Ho[1], Taira Hidaka[2], Mukhlis A Rahman[3], Naoko Yoshida[1*]

[1] Department of Civil Engineering, Nagoya Institute of Technology, Nagoya, Japan
[2] Department of Environmental Engineering, Graduate School of Engineering, Kyoto University, Kyoto University Katsura, Nishikyo, Kyoto 615-8540, Japan
[3]Advanced Membrane Technology Research Centre (AMTEC), School of Chemical and Energy Engineering (FCEE), Universiti Teknologi Malaysia, 81310 UTM Skudai, Johor, Malaysia

I. The degradation of NH_4^+ following treatment with MFCs

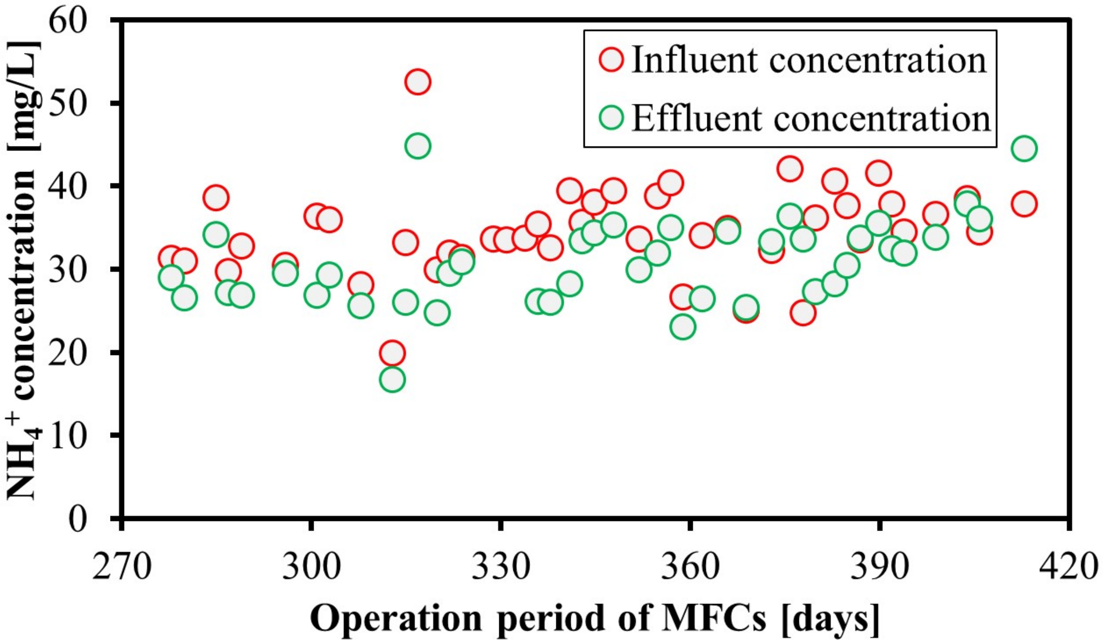

Fig S1. The degradation of NH_4^+ concentration following treatment with MFCs. The influent concentration represented the NH_4^+ concentration in sewage, while the effluent concentration indicated the NH_4^+ concentration after treatment with MFCs.

Fig S1 shows the degradation of NH_4^+ after MFCs treatment, which coincided with the period using natural zeolite adsorption and photosynthesis. The average influent concentration of NH_4^+ was approximately 35.16 mg/L, decreasing to 31.9 mg/L after passing through the MFCs. These results demonstrated that the MFCs system, which used an anion exchange membrane as a separator, was not efficient in removing NH_4^+ from sewage. Therefore, a second step was necessary for NH_4^+ removal. The integration of natural zeolite adsorption and photosynthesis clearly proved to be effective in removing NH_4^+, as shown in Fig 4(c1) and 4(c2) of the manuscript.

II. Comparison of NH_4^+ removal efficiency in this study with other reported systems

Table S1 presents the most popular combinations of MFCs with other technologies for the removal of nitrogenous compounds from wastewater. These studies achieved ammonium removal efficiencies exceeding 75%. Similar to these previous studies, our results demonstrated the effectiveness of this system in removing ammonium following MFCs treatment. Based on these review systems, the combination of MFC and membrane produced good output values. However, this approach raises several operational issues, including high costs, membrane fouling, and significant energy requirements for processes like air scouring to control bacterial growth on the

membranes (Al-Asheh, Bagheri et al. 2021, Bhattacharya and Banerjee 2023). In contrast, our system showed promise due to its low cost and ease of operation. It utilized natural zeolite, which is abundant and inexpensive (Wang and Peng 2010), and did not require additional energy for operation (e.g., LED lights).

Table S1. Comparison the NH_4^+ removal of this study and other reported systems

**Type of systems**	**Ammonium concentration [mg/L]**		**Highest removal-EC [%]**	**References**
	Inf	Eff		
MFC-Membrane bioreactor	32 ±4.28	0.8 ±0.41	97.7±1.9	(Malaeb, Katuri et al. 2013)
Regular MFC-Denitrifying MFC	21.4±10.2	4.9±3.8	77	(Zhang, Ge et al. 2013)
MFC- FO	780	114.7	85.3±3.5	(Qin, Hynes et al. 2017)
MFC-CDI	21.4	0.6±0.1	97.6	(Feng, Tsai et al. 2017)
MFC-AA/O	100-130	16-25	80.7-84	(Liu, Tursun et al. 2017)
UFCW-MFC	39	3.51	91	(Oon, Ong et al. 2015)
MFC-ZP	29.6-34.7	10.4-3.1	60-84.5	This study

Inf: influent concentration; Eff: effluent concentration; EC: efficiency; FO: forward osmotic, CDI: capacitive deionization; AA/O: anaerobic–anoxic–oxic; UFCW-MFC: up-flow constructed wetland-microbial fuel cell; ZP: natural zeolite adsorption integrated with photosynthesis.

References

Al-Asheh, S., et al. (2021). "Membrane bioreactor for wastewater treatment: A review." Case Studies in Chemical and Environmental Engineering **4**: 100109.

Bhattacharya, P. and P. Banerjee (2023). Antibacterial and Antifouling Properties of Membranes. Membranes for Water Treatment and Remediation, Springer**:** 249-276.

Feng, C., et al. (2017). "Integrating cost-effective microbial fuel cells and energy-efficient capacitive deionization for advanced domestic wastewater treatment." Chemical Engineering Journal **330**: 1-10.

Liu, R., et al. (2017). "Microbial community dynamics in a pilot-scale MFC-AA/O system treating domestic sewage." Bioresource Technology **241**: 439-447.

Malaeb, L., et al. (2013). "A hybrid microbial fuel cell membrane bioreactor with a conductive ultrafiltration membrane biocathode for wastewater treatment." Environmental science & technology **47**(20): 11821-11828.

Oon, Y.-L., et al. (2015). "Hybrid system up-flow constructed wetland integrated with microbial fuel cell for simultaneous wastewater treatment and electricity generation." Bioresource Technology **186**: 270-275.

Qin, M., et al. (2017). "Ammonium removal from synthetic wastewater promoted by current generation and water flux in an osmotic microbial fuel cell." Journal of cleaner production **149**: 856-862.

Wang, S. and Y. Peng (2010). "Natural zeolites as effective adsorbents in water and wastewater treatment." Chemical Engineering Journal **156**(1): 11-24.

Zhang, F., et al. (2013). "Long-term performance of liter-scale microbial fuel cells treating primary effluent installed in a municipal wastewater treatment facility." Environmental science & technology **47**(9): 4941-4948.
